# Synthesis, optical properties, and helical self-assembly of a bivaline-containing tetraphenylethene

**DOI:** 10.1038/srep19277

**Published:** 2016-01-13

**Authors:** Hongkun Li, Xiaoyan Zheng, Huimin Su, Jacky W. Y. Lam, Kam Sing Wong, Shan Xue, Xuejiao Huang, Xuhui Huang, Bing Shi Li, Ben Zhong Tang

**Affiliations:** 1 Key Laboratory of New Lithium-Ion Battery and Mesoporous Material, Department of Chemistry and Environmental Engineering, Shenzhen University, Shenzhen 518060, China; 2 Department of Chemistry, Institute for Advanced Study, Institute of Molecular Functional Materials, and State Key laboratory of Molecular Neuroscience, The Hong Kong University of Science & Technology, Clear Water Bay, Kowloon, Hong Kong, China; 3 Laboratory of Advanced Optoelectronic Materials, College of Chemistry, Chemical Engineering and Materials Science, Soochow University, Suzhou, Jiangsu 215123, China; 4 HKUST-Shenzhen Research Institute, No. 9 Yuexing 1st RD, South Area, Hi-tech Park, Nanshan, Shenzhen 518057, China; 5 Department of Physics, HKUST, Clear Water Bay, Kowloon, Hong Kong, China

## Abstract

A chiral tetraphenylethene derivative with two valine-containing attachments (TPE-DVAL), was synthesized by Cu(I)-catalyzed azide-alkyne “click” reaction. The optical properties and self-assembling behaviours of TPE-DVAL were investigated. The molecule is non-emissive and circular dichroism (CD)-silent in solution, but shows strong fluorescence and Cotton effects in the aggregation state, demonstrating aggregation-induced emission (AIE) and CD (AICD) characteristics. TPE-DVAL exhibits good circularly polarized luminescence (CPL) when depositing on the surface of quartz to allow the evaporation of its 1,2-dichloroethane solution. SEM and TEM images of the molecule show that the molecule readily self-assembles into right-handed helical nanofibers upon the evaporation of its solvent of DCE. The molecular alignments and interactions in assembling process are further explored through XRD analysis and computational simulation. The driving forces for the formation of the helical fibers were from the cooperative effects of intermolecular hydrogen bonding, π-π interactions and steric effect.

In the past decade, low-dimensional micro/nanostructures of organic fluorescent molecules have drawn considerable attention because they have distinctive photophysical properties and potential applications in biosensing and optoelectronic devices^1^,^2^,^3^,^4^,^5^,^6^. Unfortunately, most conventional organic fluorophores are normally highly emissive in their dilute solutions, but show weak fluorescence or even no emission in the aggregation or solid states^7^. This is due to the notorious aggregation-caused quenching (ACQ) effect, which is mainly caused by the formation of detrimental excimers or exciplexes. Since luminescent materials are most commonly used in the solid state, the ACQ effect thus limits their practical applications. In 2001, Tang’s group discovered an “abnormal” photophysical phenomenon of AIE, which was contrary to the ACQ effect^8^. AIE-active luminogens, such as silole, tetraphenylethene (TPE), and their derivatives were induced to emit intensively upon aggregation, whereas they were non-emissive when molecularly dissolved in their good solvents^9^,^10^,^11^.

Thus, AIE molecules offer great advantages as ideal building blocks for the construction of low dimensional luminescent micro/nanostructures because of their high fluorescence efficiency at solid-state and highly functional variety in the derivatives^12^,^13^,^14^,^15^,^16^,^17^,^18^,^19^. In recent years, exploring work has been carried out through this approach. Sun and Tang fabricated fluorescent one-dimensional (1D) micro/nanostructures with different morphologies, such as fibers, wires, rods and ribbons, by the self-assembling of TPE-modified fumaronitriles and perylene bisimides^20^,^21^. Zheng and coworkers synthesized AIE-active TPE-based macrocycles, which can assemble into nanofibers, microtubes and hollow microspheres in THF-water mixtures with different water fractions^22^,^23^. Han and Cho reported the construction of red fluorescent nanofibers by the self-assembling of an azobenzene derivative with AIE enhancement characteristics^24^. Ouyang *et al*. prepared a group of AIE-active phenylenediamines with tunable assemblies ranging from microblocks, microparticles, microrods to nanowires, which showed different emission colours in THF/water mixtures^25^. Among these well-defined micro/nanostructures consisting of AIE building blocks, there are few reports about the construction of helical structures^26^,^27^. Considering the importance of helicity on the optical properties of luminophores, endowing AIE building blocks with helicity is beyond doubt enriching the functions of the resultant micro/nanostructures. A simple way to induce helicity is through chiral centers, as well exemplified with naturally appeared proteins. Proteins form helical conformation and super-helical assemblies, which are induced by the chiral centres in their amino acid sequence. Inspired by the structural codes of amino acids, we have recently introduced an amino acid pendant into TPE scaffold and synthesized chiral TPE derivatives^28^,^29^. The molecules are found to assemble into helical micro/nanofibers emitting circularly polarized light. It thus indicates that chirality can be transferred to the AIE scaffold from the chiral centres of its attachments. Further illustration of the cooperative effect of luminescent scaffolds and chiral attachments is pivotal for the molecular design and functional enrichment.

In this work, we introduced two amino acids into the attachments of a TPE unit and synthesized TPE-DVAL, as shown in Fig. 1. TPE-DVAL gave no emission and CD signals in its solution, but showed intensive emission and strong Cotton effects in the aggregation state, exhibiting typical AIE and AICD effects. As a cooperative effect of the AIE and AICD, the molecule also showed CPL activity. The chirality of the two amino acid-containing attachments was also amplified in the supramolecular assembly of the molecule as the formation of predominant right-handed helical nanofibers. The packing of the molecules was also studied by XRD and simulated with molecular modeling. The driving forces for the formation of the helical fibers were from the cooperative effects of intermolecular hydrogen bonding, π-π interactions and steric effect.

## Results and Discussion

### Synthesis and Characterization

**Figure 1:**
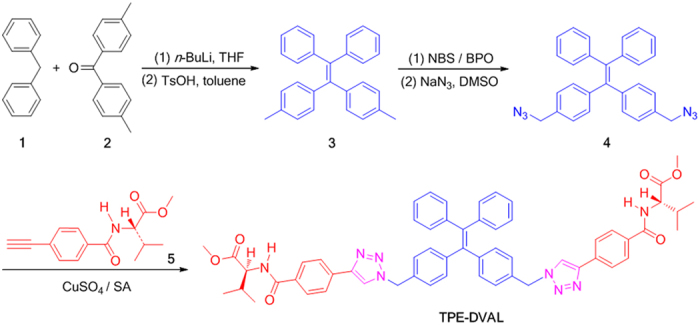
Synthetic route to TPE-DVAL. SA = sodium ascorbate.

In our previous work, we prepared a chiral TPE derivative bearing one valine attachment, which exhibited AIE, AICD and CPL properties, and readily assembled into left-handed helical nanofibers^28^. Considering that the number of substituents may influence the luminescent properties and self-assembling behaviours of the AIE molecules^30^, we thus designed and synthesized a TPE derivative with two valine attachments through the alkyne-azide click reaction (Fig. 1). The detailed synthetic procedures and characterization data are described in the experimental section and Supplementary Information. In brief, lithiation of diphenylmethane (**1**) followed by coupling with 4,4′-dimethylbenzophenone (**2**), and dehydration in the presence of TsOH led to the formation of 4,4′-(2,2-diphenylethene-1,1-diyl)bis(methylbenzene) (**3**). The subsequent bromination of **3** with NBS in the presence of BPO, and followed by reaction with sodium azide yielded the key intermediate of diazide-functionalized TPE (**4**). The target compound TPE-DVAL was obtained through “click” reaction of **4** and valine-containing alkyne (**5**) catalyzed by CuSO_4_/sodium ascorbate^31^. All the reactions proceeded smoothly. The structures of the intermediates and product were characterized by standard spectroscopic methods and elemental analysis, from which satisfactory data were obtained (experimental section and Supplementary Figure S1–S3).

### UV Absorption and CD Spectra

**Figure 2:**
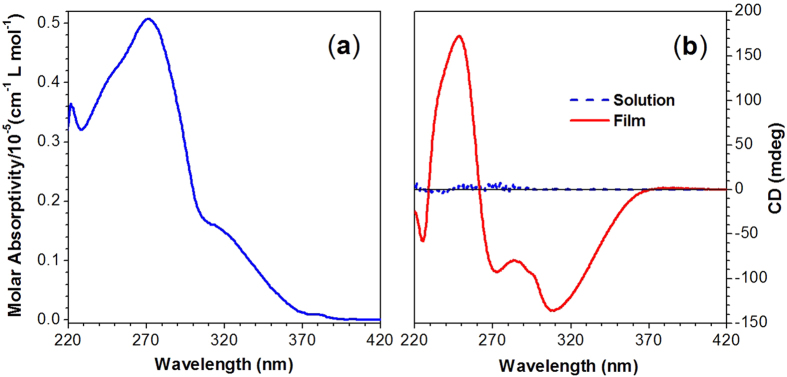
(**a**) Absorption spectrum of TPE-DVAL in 1,2-dichloroethane (DCE). (**b**) CD spectra of TPE-DVAL solution (in DCE, concentration: 0.5 mg mL^−1^) and film prepared by natural evaporation of its DCE solution (concentration: 0.5 mg mL^−1^) on a quartz substrate.

The UV spectrum of TPE-DVAL in 1,2-dichloroethane (DCE) solution (Fig. 2a) exhibits two absorption peaks at the wavelength of 318 and 271 nm with molar absorptivities of 1.49 × 10^4^ and 5.06 × 10^4^ L mol^−1^ cm^−1^, respectively. They correspond to the absorptions of the TPE core and the peripheral triazolylphenyl groups, respectively. The CD spectra of TPE-DVAL are given in Fig. 2b. The compound barely shows signals in DCE solution^32^, but exhibits strong Cotton effects at the wavelength of 249, 272 and 310 nm after depositing on the surface of quartz to allow natural evaporation of its DCE solution, which is a typical AICD effect^33^. The peaks at 249 and 272 nm are associated with the absorptions of triazolylphenyl groups, while the negative Cotton effect at 310 nm is considered to stem from the absorption of TPE cores. This AICD effect implies that in the aggregation the TPE scaffolds are helically arranged with a preferred screw sense, which is induced by the l-valine-containing attachments.

### Aggregation-Induced Emission

**Figure 3:**
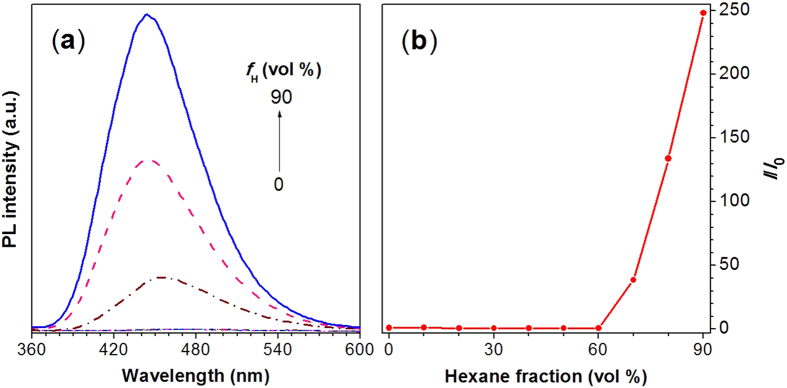
(**a**) PL spectra of TPE-DVAL in pure DCM and DCM/hexane mixtures with different volume fractions of hexane (*f*_H_). Concentration: 10 μM. λ_ex_: 320 nm. (**b**) Plot of relative emission peak intensity (*I/I*_0_) at 444 nm versus hexane fraction of the DCM/hexane mixtures, where *I* = peak intensity and *I*_0_ = peak intensity in pure DCM.

The PL spectra of TPE-DVAL in the solution and aggregate states were investigated. Since it was soluble in DCM, but insoluble in hexane, we then chose hexane as the poor solvent to induce the aggregation and measured the emission spectra of TPE-DVAL in DCM/hexane mixtures with different hexane fractions (*f*_H_). As can be seen from Fig. 3, the PL curves of TPE-DVAL in DCM and DCM/hexane mixtures with *f*_H_ lower than 60% barely showed emission. The PL intensity started to increase at the *f*_H_ higher than 60% and reached the highest intensity at the *f*_H_ of 90%, which is 248 times higher than that in its DCM solution (Fig. 3b). As hexane is the poor solvent for TPE-DVAL, the addition of hexane into its DCM solution at high percentage (*f*_H_ > 60%) would induce the aggregation of the molecules. The aggregation thus restricted the intramolecular motions of TPE-DVAL, which blocked the non-radiative decay channels and turned on its fluorescence^11^, showing the typical AIE effect.

### Circularly Polarized Luminescence

CPL, the anisotropic emission of circularly polarized light from chiral molecular systems, can provide specific information about the chirality of the molecules. In recent years, small organic molecules with CPL property have attracted increasing attention due to their structural diversity and potential applications in chiroptical sensing, optical information processing and storage, and light-emitting devices^34^,^35^,^36^,^37^. The performance of CPL-active materials is generally evaluated by the emission dissymmetry factor (*g*_em_), defined as *g*_em_ = 2(*I*_L_ − *I*_R_)/(*I*_L_ + *I*_R_), where *I*_L_ and *I*_R_ denote the emission intensities of left- and right-handed circularly polarized light, respectively. Most of the chiral fluorophores, such as helicene, binaphthalene, and their derivatives, show the absolute *g*_em_ in the range of 10^−5^–10^−2^ in solutions^38^,^39^,^40^,^41^,^42^,^43^,^44^. Their CPL performance at solid state, however, usually becomes even worse due to the intrinsic ACQ effect. Among the limited number of chiral AIE molecules reported^26^,^27^,^28^,^29^, few organic functional materials have high CPL activity at solid state.

**Figure 4:**
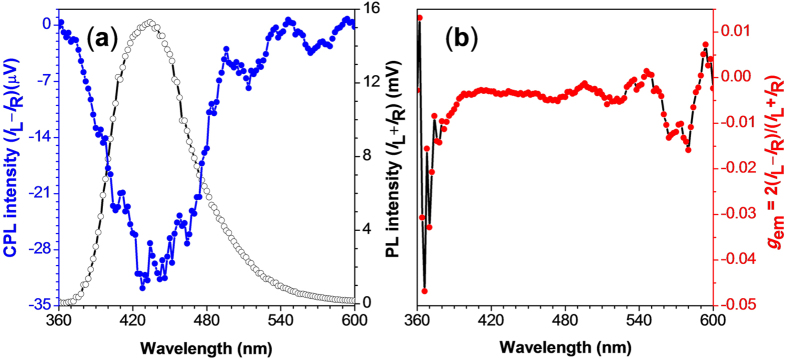
Plots of (**a**) CPL and PL and (**b**) CPL dissymmetry factor (*g*_em_) *versus* wavelength of cast film of TPE-DVAL formed by evaporation of its DCE solution.

Because TPE-DVAL has both AICD and AIE property, we then further explore whether these two properties combined cooperatively to generate CPL properties. Since TPE-DVAL was CD-silent and non-emissive in solutions, we thus only measured the CPL spectra of TPE-DVAL at the solid state using a home-built CPL spectrometer^26^. The dependence of CPL, PL and *g*_em_ on the wavelength was shown in Fig. 4. The solid film of TPE-DVAL formed by natural evaporation of its DCE solution gave negative signals in the CPL spectrum (Fig. 4a), which was corresponding well with its CD spectrum. This further indicated that the fluorophores adopted helical conformations in the aggregation state with a preferred screw sense. The *g*_em_ values were in the range of −3.5 × 10^−3^ to −5.2 × 10^−3^ at the wavelength of 400–520 nm (Fig. 4b), which were comparable to those of most reported CPL-active materials (|*g*_em_| = 10^−5^–10^−2^)^38^,^39^,^40^,^41^,^42^,^43^,^44^. While considering that TPE-DVAL does not suffer from ACQ effect that normal fluorescent molecules suffer, TPE-DVAL is certainly a promising candidate for high-tech applications.

### Self-Assembly and Structural Modeling

**Figure 5:**
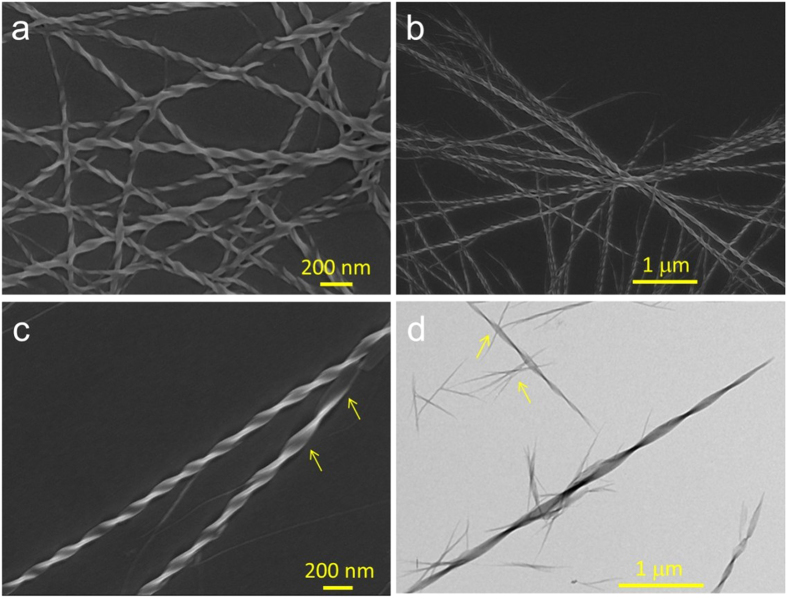
(**a–c**) SEM and (**d**) TEM images of TPE-DVAL formed by natural evaporation of its DCE solution, concentration: 1 × 10^−4^ M.

In nature, proteins easily self-assemble into helical structures via non-covalent interactions of their amino acids. When amino acid attachment is introduced to the TPE scaffold, the chirality of the amino acid is expected to transfer to the AIE scaffold and amplified in their supramolecular assemblies. Indeed, our previous work has proved this expectation. The TPE derivative bearing one valine-substituent, TPE-VAL, can readily self-assemble into left-handed nanofibers in DCE/hexane (1/9, v/v) mixture^28^. With respect to TPE-DVAL, the TPE derivative carrying two valine substituents, is also anticipated to form helical nanostructures. We thus investigated the self-assembling behaviours of TPE-DVAL using SEM and TEM techniques. It was found that the molecules readily assembled into nanofibers with the predominantly right-handedness in DCE upon solvent evaporation (Fig. 5). The helical nanofibers interlace each other. Their average width and helical pitch are ~30–70 and ~100–200 nm, respectively (Fig. 5b). Their length can be up to several micrometers, as clearly seen in Fig. 5a. The combined structures showing both the morphology of helical ribbons and fibers were also observed (labelled with arrows in Fig. 5c,d), which indicates that the helical nanofibers are likely formed by the wrapping up of the nanoribbons. Though both TPE-DVAL and TPE-VAL self-assemble into helical fibers, the helical fibers differ obviously. Besides the opposite dominant handedness, they also differ in their appearance and TPE-DVAL has more uniform twisting angles. These results suggest that a change of the number of amino acid attachments can lead to a diverse change in their assembling behaviour and accordingly their optical activities.

**Figure 6:**
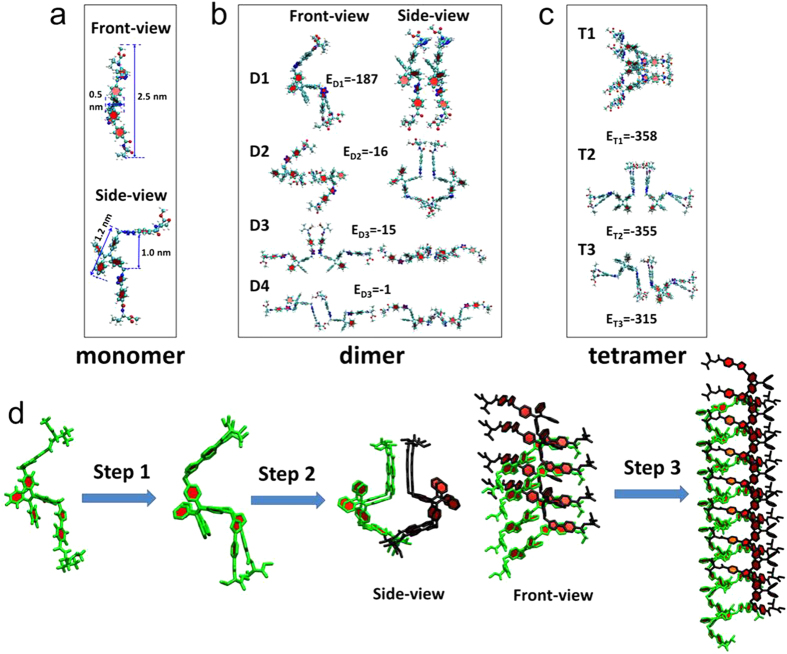
Optimized (**a**) monomer, (**b**) dimers, (**c**) tetramers structures based on AM1//HF/6-31G* method in Gaussian 09 program, and (**d**) energy minimized nanofiber structures of TPE-DVAL. (**a**) optimized monomer structure in different views; (**b**) optimized dimers **D1**, **D2**, **D3** and **D4** in both front- and side-view with binding energy (in kJ/mol) below each structure; (**c**) optimized tetramers **T1**, **T2**, and **T3** with binding energy (in kJ/mol) below each structure. (**d**) Proposed assembly flows of TPE-DVAL: single column packing (step1), bi-column association (step 2); longitudinal elongation of bi-column association (step 3).

**Figure 7:**
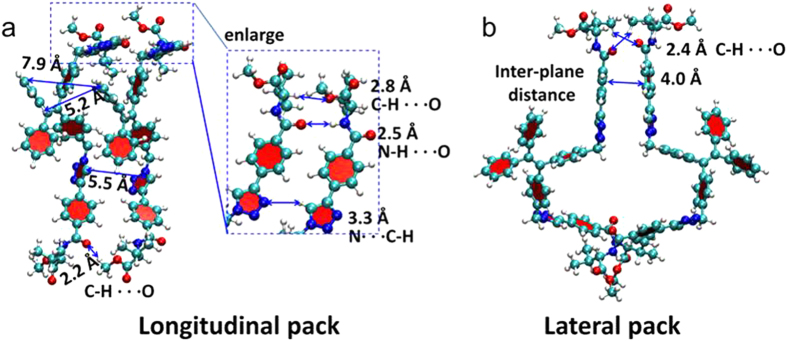
Structural analysis of the intermolecular interactions in the energy-favored (**a**) longitudinal and (**b**) lateral pack models, which are extracted from dimer **D1** and **D2** in Fig. 6, respectively.

In order to decipher the supramolecular assembling behaviour of TPE-DVAL, we employed powder X-ray diffraction (XRD) technique and molecular modeling. The XRD data indicated that there were characteristic d-spacings of ~2.5 nm, 0.79–1.25 nm, and ~0.4–0.5 nm (Supplementary Figure S4) in the molecular packing. To correlate these characteristic spacings with the molecular structures, molecular modeling was also carried out to model different possible packing modes. As displayed in Fig. 6, the structures of the monomer, dimers and tetramers of TPE-DVAL were optimized in different packing modes through quantum chemical calculations using AM1//HF/6-31G* method in Gaussian 09 program^45^. According to the optimization, the monomer has a persistent length of ~2.5 nm spanning two side-chains (Fig. 6a). With respect to the different packing modes of the dimers, apparently when the TPE scaffolds stack in a face to face manner, the resultant dimer has the lowest potential energy (**D1**, Fig. 6b). The distance between the two neighboring stacks in **D1** is 7.9 Å and the corresponding π-π distance is ~5.2 Å (Fig. 7), which is beyond the interaction distance of π-π stacking. This is consistent with the absence of induced CD signal at the long wavelength by excitonic coupling. Two dimers, either with the same or different packing mode could further associate together to form tetramers. As shown in Fig. 6, dimer **D1** could associate with dimer **D2**, **D3** or **D4** in a side by side way to form tetramer **T1**, **T2** or **T3**, respectively (Fig. 6c). As **T1** formed a helical-like structure with the lowest energy, it should overwhelm its open-armed counterpart tetramers **T2** and **T3**. The energy differences between **T1**, **T2** and **T3** are smaller than the corresponding energy differences between different dimers, suggesting that the total energy of the oligomers decreased due to the cooperative effect. Combining the results of molecular modeling and experimental XRD data together, we propose the packing diagram for the self-assembling process (Fig. 6d).

The molecules tend to pack in a face to face manner to elongate longitudinally, forming a column (step 1 in Fig. 6d); two columns further associate in a side by side way to grow laterally (step 2 in Fig. 6d). The chirality and steric effect of the amino acids will result in a slight dislocation between each stack and the dislocation will be amplified as a significant twist with more molecules joining in the packing. As a combinational effect of the elongation and lateral growth of columns, helical nanofibers and nanoribbons will be formed (step 3 in Fig. 6d) as evidenced with the assemblies in SEM and TEM images. The molecular modeling results agree well with the XRD results, the d-spacings of 2.5 and 0.79 nm are suggested to correspond with the width and helical unit height of a single stack of the helical structures, respectively.

To understand the underlying principle of the assembling process, we also explored the main driving force for the assembling process. Two representative dimers **D1** and **D2** are selected for further analysis to identify the favorable interactions that may result in the longitudinal and lateral growth of nanofibers. We found that several patterns of hydrogen bonds, such as C-H…O (~2.8 Å and 2.2 Å), N-H…O (~2.5 Å) and C-H…N (~3.3 Å), coexist in the side chains of dimer **D1**. The π-π stacking distance between two parallel aromatic groups of amino acid attachments is 5.5 Å and the average distance between neighbouring TPE stacks of the column is 5.2 Å (Fig. 7a). With respect to dimer **D2**, there are two weak pairs of hydrogen bonds C-H…O (~2.4 Å) and one weak π-π interaction with corresponding π-π stacking distance of ~4.0 Å (Fig. 7a). We anticipate that these hydrogen bonds in multiple interaction sites and weak π-π interactions may collectively stabilize dimer **D1**, and thus make it more favorable to form than dimer **D2** and **D3**. There is also steric effect of the amino acid attachments involved in the assembling process. In summary, the driving force in the self-assembling process comes from the cooperative effects from the multiple intermolecular hydrogen bonding interactions (N-H…O, C-H…O, C-H…N), π-π interactions, and also the compromise of the steric effect between the attachments. The detailed assembling mechanism is still under investigation in our laboratories.

The introduction of chirality into AIE scaffold not only adds new functions to AIE molecules, but also offers the possibility to design novel architectures biomimetically with AIE building blocks. The cooperative effect of the chiral attachments and AIE scaffold is pivotal in determining the self-assembled architectures and the output of their optical property. Because of the highly sensitivity of the cooperative effect of the noncovalent interactions, this kind of molecules have important potentials in the applications of biosensing and chemosensing. The underlying mechanism that governs the assembling process and the optical properties of the architectures are worth further exploring due to their great importance for the structure design and function optimization.

## Conclusions

In summary, we have designed and synthesized TPE-DVAL which has TPE scaffold and two valine attachments through the azide-alkyne click reaction. TPE-DVAL was CD-silent and non-emissive in solution, but gave strong CD signals and emit intensely in the aggregation state, showing AICD and AIE characteristics. The compound was also aggregation-induced CPL-active. Its film preferred to emit right-handed circularly polarized light and exhibited good CPL performance (|*g*_em_| ≈ 3.5–5.2 × 10^−3^). It readily self-assembled into predominantly right-handed helical nanofibers upon the evaporation of its DCE solution. Computational simulation was employed to clarify how the molecules were packed and arranged in the self-assembling process. The driving force for the assembling came from a cooperative effect of the synergistic multiple intermolecular weak hydrogen bonding, π-π interactions, and steric effect. TPE-DVAL is of promising application for fabricating efficient CPL devices for bio-sensing and optoelectronic applications.

## Methods

### Materials

Dichloromethane (DCM) was distilled over calcium hydride in an atmosphere of nitrogen immediately prior to use. Hexane and tetrahydrofuran (THF) were distilled from sodium benzophenoneketyl in nitrogen atmosphere just prior to use. *p*-Toluenesulfonic acid monohydrate (TsOH), *N*-Bromosuccinimide (NBS), benzoyl peroxide (BPO), l-Ascorbic acid, and other chemicals and solvents were all purchased from Aldrich and used as received without further purification.

### Instruments

^1^H and ^13^C NMR spectra were recorded on a Bruker ARX 400 NMR spectrometer in CDCl_3_ or DMSO-*d*_6_ using tetramethylsilane (TMS; *δ* = 0) as internal reference. High-resolution mass spectrum (HRMS) was taken on a GCT Premier CAB 048 mass spectrometer in a MALDI-TOF mode. Elemental analysis was measured on a Thermo Finnigan Flash 1112 system. UV absorption spectra were recorded on a Milton Ray Spectronic 3000 array spectrophotometer. Photoluminescence (PL) spectra were taken on a Perkin-Elmer LS 55 spectrofluorometer. Morphologies of the aggregates were examined by JEOL 2010 transmission electron microscope (TEM) at an accelerating voltage of 200 kV and JEOL-6700F scanning electron microscope (SEM). CD spectra were measured on a JASCO J-810 spectropolarimeter in a 1 mm quartz cuvette using a step resolution of 0.1 nm, a scan speed of 100 nm/min, a sensitivity of 0.1 nm, and a response time of 0.5 s. Circularly polarized photoluminescence spectra were taken on a home-made CPL spectroscopy system (shown in Supplementary Figure S5)^26^. The excitation light source is 325 nm He-Cd laser. The retardation of the emitted light from the sample is modulated through a photo-elastic modulator (PEM; Hinds PEM-90, 50 kHz) and detected by the photomultiplier tube (PMT) after passing through the linear polarizer oriented at 45° to the PEM optical axis. The combination of PEM and the linear polarizer provides modulation of the circularly polarized part of the total emission. The DC component of the PMT output is recorded by a digital multimeter (Thurlby 1905a), where the total intensity of left (*I*_L_) and right (*I*_R_) of circularly polarized emitted light can be obtained (i.e., *I*_L_ + *I*_R_). On the other hand, the AC component is amplified by a pre-amplifier (Stanford Research Systems, SR560) and analyzed by a lock-in amplifier (Stanford Research Systems, SR510), so that alternating signals regarding to emitted left and right polarization is detected (i.e., *I*_L_ − *I*_R_). The CPL dissymmetry factor, *g*_em_ = 2(*I*_L_ − *I*_R_)/(*I*_L_ + *I*_R_), was then calculated from the ratio of AC signal to the DC signal.

### Sample Preparation for PL Measurement

A stock DCM solution of TPE-DVAL (1 × 10^−4 ^M) was first prepared. Aliquots of the stock solution were transferred to 10 mL volumetric flasks. After appropriate amounts of DCM were added, hexane, the poor solvent, was added dropwise under vigorous stirring to give 1 × 10^−5 ^M solutions with different hexane fractions (0–90 vol%). The PL measurements of the resulting solutions were then performed immediately.

### Sample Preparation for SEM, TEM

A DCE solution of TPE-DVAL (1 × 10^−4 ^M) was first prepared. 4 μL of the solution was then dropped onto the surface of silicon wafer (5 × 5 mm) and carbon-coated copper grid, respectively. After overnight evaporation of the solvent under ambient conditions, the samples were characterized by SEM and TEM, respectively.

### Sample Preparation for CPL measurements

A DCE solution of TPE-DVAL (1 mg mL^−1^) was first prepared. 1 mL of the DCE solution of TPE-DVAL (1 mg mL^−1^) was deposited dropwise onto the quartz plates with a diameter of 2 cm. After overnight evaporation of the solvent under ambient conditions, the samples were characterized by CPL.

### Synthesis

4-Ethynylbenzoyl-*l*-valine methyl ester (5) was prepared according to our previous paper^46^. TPE-DVAL was synthesized through multistep reactions as shown in Fig. 1. The detailed synthetic procedures for the reaction intermediates are given in the Supplementary Information. The procedures for the synthesis of TPE-DVAL are described below.

### Synthesis of TPE-DVAL

Into a 100 mL two-necked flask were added 0.222 g (0.5 mmol) of 4,4′-(2,2-diphenylethene-1,1-diyl)bis[(azidomethyl)benzene] (**4**) and 0.285 g (1.1 mmol) of 4-ethynylbenzoyl-l-valine methyl ester (**5**) in 30 mL of water/THF/ethanol (1/1/1, v/v/v) under nitrogen. Freshly prepared 1 M aqueous solution of sodium ascorbate (0.63 mL) was added, followed by12 mg (0.075 mmol) of copper sulfate in 0.5 mL of water. The reaction mixture was stirred at 70 °C overnight. After cooling to room temperature, 60 mL of water was added and the resultant solution was extracted with DCM. The combined organic layer was washed with brine, ethylene diamine tetraacetic acid disodium salt aqueous solution, and water. After solvent evaporation, the crude product was purified by a silica gel column using DCM/ethyl acetate (4: 1 by volume) mixture as eluent. A white solid was obtained with a yield of 44.0%. ^1^H NMR (400 MHz, DMSO-*d*_6_), *δ* (TMS, ppm): 8.68 (s, 2 H), 8.66-8.64 (d, 2 H), 7.97-7.90 (m, 8 H), 7.14 − 7.09 (m, 10 H), 7.08 − 6.94 (m, 8 H), 5.50 (s, 4 H), 4.31 − 4.27 (m, 2 H), 3.65 (s, 6 H), 2.21 − 2.16 (m, 2 H), 0.99 − 0.93 (m, 12 H). ^13^C NMR (100 MHz, DMSO-*d*_6_), *δ* (TMS, ppm): 172.3, 166.6, 145.8, 143.0, 141.4, 139.4, 134.0, 133.5, 133.0, 131.1, 130.6, 128.4, 127.9, 127.5, 126.8, 124.8, 122.5, 58.8, 52.7, 51.7, 29.6, 19.2. HRMS (m/z): [M + Na]^+^ calcd. for C_58_H_56_NaN_8_O_6_, 983.4221; found, 983.4247; anaysis (calcd., found for C_58_H_56_N_8_O_6_): C (72.48, 72.23), H (5.87, 5.85), N (11.66, 11.44).

## Additional Information

**How to cite this article**: Li, H. *et al*. Synthesis, optical properties, and helical self-assembly of a bivaline-containing tetraphenylethene. *Sci. Rep.* **6**, 19277; doi: 10.1038/srep19277 (2016).

## Supplementary Material

Supplementary Information
