# Supplementary material for: Synthesis, optical properties, and helical self-assembly of a bivaline-containing tetraphenylethene
Source: Sci Rep. 2016 Jan 13;6:19277. doi: 10.1038/srep19277 (PMC4725923; doi:10.1038/srep19277)
Supplement: Supplementary Information [file srep19277-s1.doc]

*Supplementary Information*

**Synthesis, optical properties, and helical self-assembly of a bivaline-containing tetraphenylethene**

Hongkun Li,^1,2,3^ Xiaoyan Zheng,^2^ Huimin Su,^5^ Jacky W. Y. Lam,^2,4^ Kam Sing Wong,^5^

Shan Xue, ^1^ Xuejiao Huang, ^1^ Xuhui Huang,^2^ Bing Shi Li^1^,*& Ben Zhong Tang^2,4^,*

*^1^Key Laboratory of New Lithium-Ion Battery and Mesoporous Material, Department of Chemistry and Environmental Engineering, Shenzhen University, Shenzhen 518060, China. E-mail: phbingsl@szu.edu.cn; Fax: 86-755-26536141; Tel: 86-755-26558094*

*^2^Department of Chemistry, Institute for Advanced Study, Institute of Molecular Functional Materials and State Key Laboratory of Molecular Neuroscience, The Hong Kong University of Science & Technology (HKUST), Clear Water Bay, Kowloon, Hong Kong, China. E-mail: tangbenz@ust.hk*

*^3^Laboratory of Advanced Optoelectronic Materials, College of Chemistry, Chemical Engineering and Materials Science, Soochow University, Suzhou, Jiangsu 215123, China*

*^4^HKUST-Shenzhen Research Institute, No. 9 Yuexing 1st RD, South Area, Hi-tech Park, Nanshan, Shenzhen 518057, China*

*^5^Department of Physics, HKUST, Clear Water Bay, Kowloon, Hong Kong, China*

**Table of Contents**

Synthetic procedure for the reaction intermediate **4**. S2

**Figure S1.** ^1^H NMR spectrum of TPE-DVAL in DMSO-*d*_6_. S3

**Figure S2.** ^13^C NMR spectrum of TPE-DVAL in DMSO-*d*_6_. S3

**Figure S3.** High resolution mass spectrum of TPE-DVAL. S4

**Figure S4.** XRD diffractogram of the powder of TPE-DVAL prepared by natural evaporation of its DCE solution. S4

**Figure S5.** Optical setup for the CPL measurement. S5

**Preparation of 4,4'-(2,2-diphenylethene-1,1-diyl)bis(methylbenzene) (3)**. Into a 250 mL two-necked round-bottom flask was added 4.374 g (26 mmol) of diphenylmethane. The flask was evacuated and refilled with dry nitrogen three times. 60 mL of distilled THF was added to dissolve the solid. A solution of *n*-butyllithium (12 mL, 24 mmol) in hexane was injected at 0 °C. The resulting solution was stirred at °C for 30 min. To the solution was then added 4.205 g (20 mmol) of 4,4ʹ-dimethylbenzophenone dissolved in 40 mL of distilled THF. After stirring for 6 h at room temperature, the reaction was quenched with an aqueous solution of NH_4_Cl. The resultant solution was extracted with DCM. The combined organic layer was washed with brine and dried over MgSO_4_. After filtration and solvent evaporation, the crude product was dissolved in 100 mL of distilled toluene in a 250 mL flask fitted with a Dean Stark trap. 0.761 g (4 mmol) of *p*-toluenesulfonic acid (TsOH) was added and the mixture was refluxed overnight. After cooling to room temperature, the reaction mixture was washed with 10% aqueous NaHCO_3_ solution and the combined organic layer was dried over MgSO_4_. After filtration and solvent evaporation, the crude product was purified by a silica gel column using petroleum ether as eluent to give a white solid in 84.5% yield (6.094 g). ^1^H NMR (CDCl_3_, 400 MHz), *δ* (TMS, ppm): 7.08 (m, 6H), 7.03 (m, 4H), 6.90 (m, 8H), 2.25 (s, 6H). ^13^C NMR (CDCl_3_, 100 MHz), *δ* (TMS, ppm): 144.3, 141.1, 141.0, 140.2, 136.1, 131.5, 131.4, 128.5, 127.8, 126.4, 21.4.

**Preparation of 4,4'-bis(azidomethyl)tetraphenylethene (4)**. Into a 250 mL round-bottom flask was added 5.191 g (14.4 mmol) of **3**. 150 mL of carbon tetrachloride was injected to dissolve the solid under nitrogen and the mixture was heated to reflux. 5.382 g (30.24 mmol) of N-bromobutanimide (NBS) and 0.035 g (0.144 mmol) of benzoyl peroxide (BPO) were added in portions and the reaction mixture was refluxed overnight. After cooling to room temperature, the inorganic salts were removed by filtration. After solvent evaporation, the obtained crude product was dissolved in 150 mL of dimethyl sulfoxide (DMSO), and followed by addition of 2.808 g (43.2 mmol) of sodium azide. The reaction mixture was stirred at 60 °C overnight. After cooling, a large amount of water was added. The resulting solution was extracted with diethyl ether. The combined organic layer was washed with water and brine, and dried over MgSO_4_ overnight. After filtration and solvent evaporation, the crude product was purified by a silica gel column using petroleum ether-ethyl acetate (100 : 1, v/v) as eluent. A white solid of **4** was obtained with a yield of 75.5%. ^1^H NMR (CDCl_3_, 400 MHz), *δ* (TMS, ppm): 7.10 (m, 6H), 7.03 (m, 7H), 7.00 (m, 5H), 4.25 (s, 4H). ^13^C NMR (CDCl_3_, 100 MHz), *δ* (TMS, ppm): 143.7, 143.4, 142.1, 139.7, 137.7, 133.5, 132.5, 131.9, 131.8, 131.4, 130.2, 128.4, 127.8, 126.9, 126.8, 125.9, 54.6.

**Scheme S1** Synthetic route to **4**.

**Figure S1.** ^1^H NMR spectrum of TPE-DLVL in DMSO-*d*_6_. The solvent and water peaks are marked with asterisks.

**Figure S2.** ^13^C NMR spectrum of TPE-DVAL in DMSO-*d*_6_. The solvent peaks are marked with asterisk.


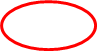


983.4247

**Figure S3.** High resolution mass spectrum of TPE-DVAL.

**Figure S4.** XRD diffractogram of the powder of TPE-DVAL prepared by natural evaporation of its DCE solution.


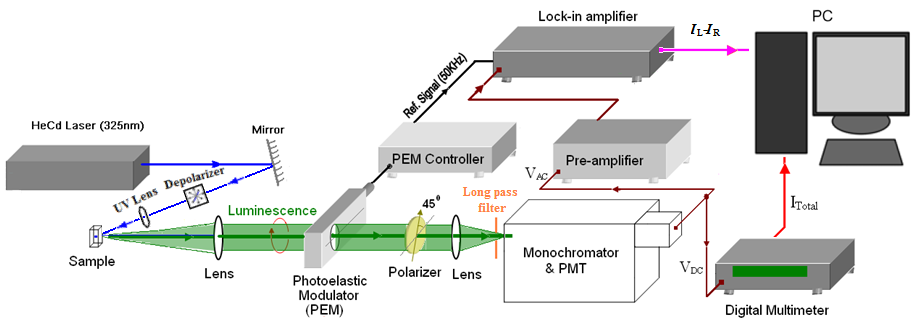


**Figure S5.** Optical setup for the CPL measurement.
